# Supplementary material for: Patterns of Intron Gain and Loss in Fungi
Source: PLoS Biol. 2004 Nov 30;2(12):e422. doi: 10.1371/journal.pbio.0020422 (PMC532390; doi:10.1371/journal.pbio.0020422)
Supplement: Table S1 — Also available at http://genes.mit.edu/NielsenEtAl/. (4.3 MB ZIP). [file pbio.0020422.st001.zip › NielsenEtAl/html/1401.html]

AN4470.1.NCU02810.1.MG01592.1.FG06843.1


```
 CLUSTAL W (1.82) Multiple Sequence Alignments - Introns Inserted


Sequence 1: NCU02810.1	511 aa
Sequence 2: MG01592.1	504 aa
Sequence 3: FG06843.1	509 aa
Sequence 4: AN4470.1	514 aa
Alignment Length: 516 aa
Number Identitical Residues: 361 aa
Alignment Score (without introns) 15268


MG01592.1 	-MSSGDKFGNDIESESDSGPDSPGLDPQDHSDGEIDEKPLKSALKKT--VVAPPPIQRPE
NCU02810.1	MATNGDKFGNDIV-ESDSGSDNESHVDQHESDNEVEAKPRKSALKK----APAPVVQRPP
FG06843.1 	MSTNDDPKYDDIGSESDSG-ESVGHN------EAADEKPLKSALKKSNPAIAEPAAQRPP
AN4470.1  	MATNGDFSDEESQPGSPILNANGQDDIQDQEPLEQEEKPIKSAMKKD--FIPVSQPKRPE
          	  :..*   ::  . *     .     .  .    : ** ***:**     . .  :** 

MG01592.1 	LPPQTDPKDLDVSKLNPLTPEIIARQATINIGTIGHVAHGKSTVVKAISGVQTVRFKNEL
NCU02810.1	LPPQTDPKDLDVKTLSPLTPEIIARQATINIGTIGHVAHGKSTVVKAISGVQTVRFKNEL
FG06843.1 	LPPQTEPKDLDVASLTPLTPEIIARQATINIGTIGHVAHGKSTVVKAISGVQTVRFKNEL
AN4470.1  	LPEQPNPETLDLSTLTPLSPEIIARQATINIGTIGHVAHGKSTVVKAISEVQTVRFKNEL
          	** *.:*: **: .*.**:****************************** **********

MG01592.1 	IRNITIKLGYANAKIYRCNNPKCPRPTAFRSYKSDKEVDPPCEREGCEGKYELLRHVS2F
NCU02810.1	IRNITIKLGYANAKIYKCDNPACPRPTCYRSYKSEKEVDPPCEREGCEGTYRLLRHVS2F
FG06843.1 	IRNITIKLGYANAKIYKCDNQACPRPGCYRSYKSDKEVDPPCERDGCGGTYRLLRHVS2F
AN4470.1  	ERNITIKLGYANAKIYKCDNPACPRPTCYKSYKSEKEIDPPCERDGCSGRYRLLRHVS2F
          	 ***************:*:*  **** .::****:**:******:** * *.****** *

MG01592.1 	VDCPGHDILMSTMLSGAAVMDAALLLIAGNESCPQPQTSEHLAAIEIMKLENIIILQNKV
NCU02810.1	VDCPGHDILMSTMLSGAAVMDAALLLIAGNESCPQPQTSEHLAAIEIMKLDKIIILQNKV
FG06843.1 	VDCPGHDILMSTMLSGAAVMDAALLLIAGNESCPQPQTSEHLAAIEIMKLDKIIILQNKV
AN4470.1  	VDCPGHDILMSTMLSGAAVMDAALLLIAGNETCPQPQTSEHLAAIEIMKLSHIIILQNKV
          	*******************************:******************.:********

MG01592.1 	DLMREEAAQQHYQSILKFIRGTVAGKSPIIPISAQLKFNVDAVVEAIVNTVPVPPRDFSV
NCU02810.1	DLMREEAAKQHYESILKFIRGTVAGKSPIIPISAQLKFNIDAINDAIVNTIPVPPRDFSM
FG06843.1 	DLMREEAAQQHYQSILKFIRGTVAGKSPVIPISAQLKFNIDAVNEAIVNTIPVPPRDFSI
AN4470.1  	DLMREDGALQHYQSILKFIRGTVADGSPIIPISAQLKYNIDAVNEYLVSHIPVPVRDFTA
          	*****:.* ***:***********. **:********:*:**: : :*. :*** ***: 

MG01592.1 	DPQMIVIRSFDVNKPGAEIDDLKGGVAGGSILHGILKLGDEIEIRPGIVTRDDKGDLQCT
NCU02810.1	DPQMIVIRSFDVNKPGAEIDELKGGVAGGSILHGVLKLGDEIEIRPGIVTRDEKGDLKCT
FG06843.1 	DPHMIVIRSFDVNKPGSEIDELKGGVAGGSILHGVIKLGDEIEIRPGIVARDDNGALKCT
AN4470.1  	SPHMIVIRSFDVNKPGAEIDELKGGVAGGSILTGVLKLNDEIEIRPGLVTKDENGKIQCR
          	.*:*************:***:*********** *::**.********:*::*::* ::* 

MG01592.1 	PIFSRIVSLNSEANELKYAVPGGLIGVGTRIDPTLCRADRLVGHVLGLKGRLPDIYSEIE
NCU02810.1	PIFSRIVSLNSEANDLKYAVPGGLIGVGTRIDPTLCRADRLVGFVLGLKGRLPEIYSEIE
FG06843.1 	PIFSRVVSLNSEANDLKYAVPGGLIGVGTRIDPTLCRADRLVGFVLGLKGRLPEIYSEIE
AN4470.1  	PIFSRVVSLFAEHNDLKFAVPGGLIGVGTRVDPTLCRADRLVGFVLGHRGRLPAIYTELE
          	*****:*** :* *:**:************:************.*** :**** **:*:*

MG01592.1 	VNFYLLRRLLGVRTADGKQAKVEKLQKNEVIMVNIGSTSTGAKVSAIKKDAAKLILTSPA
NCU02810.1	VNFYLLRRLLGVRTADGKQAKVDKLAKNEVIMVNIGSTSTGAKVSAIKKDAAKLILTSPA
FG06843.1 	VNFYLLRRLLGVRTADGKQAKVAKLAKNEVIMVNIGSTSTGAKVAAIKNDAAKLVLTSPA
AN4470.1  	VNYFLLRRLLGVKTADGKQAKVAKLAKNEVLMVNIGSTATGAKVVGVKADAAKLSLTSPA
          	**::********:********* ** ****:*******:***** .:* ***** *****

MG01592.1 	CTNIGEKVALSRRIEKHWRLIGWATIAA~---------
NCU02810.1	CTSIGEKVALSRRIEKHWRLIGWATIAA2GVTLEPSTS
FG06843.1 	CTNIGEKVALSRRIEKHWRLIGWATIAA2GVTLEPSTS
AN4470.1  	CTEVGEKIAISRRIEKHWRLIGWANIVA2GNTLEPIVN
          	**.:***:*:**************.*.* . : ..  .
```
